# Spontaneous thrombosis of a giant aneurysm complicated with the coronary-to-pulmonary artery fistula: a case report

**DOI:** 10.1093/ehjcr/ytae227

**Published:** 2024-04-26

**Authors:** Tomonori Takahashi, Tetsuzo Wakatsuki, Takayuki Ise, Masataka Sata

**Affiliations:** Department of Cardiovascular Medicine, Tokushima University Hospital, 2-50-1 Kuramoto, 770-0042 Tokushima, Japan; Department of Cardiovascular Medicine, Tokushima University Hospital, 2-50-1 Kuramoto, 770-0042 Tokushima, Japan; Department of Cardiovascular Medicine, Tokushima University Hospital, 2-50-1 Kuramoto, 770-0042 Tokushima, Japan; Department of Cardiovascular Medicine, Tokushima University Hospital, 2-50-1 Kuramoto, 770-0042 Tokushima, Japan

**Keywords:** Coronary-to-pulmonary artery fistulas, Coronary anomalies, Coil embolization, Case report

## Abstract

**Background:**

A coronary-to-pulmonary artery fistula (CPAF) with a giant aneurysm is a rare clinical occurrence. The rupture of an aneurysm leads to a fatal outcome, thus prompting the incorporation of prophylactic measures, which have encompassed surgical resections or endovascular embolization procedures. The indications for these treatment strategies are controversial, and little has been elucidated regarding the salient characteristics underpinning the selection of a therapeutic strategy. We report a case of a giant aneurysm associated with CPAFs that was thrombosed before interventional treatment.

**Case summary:**

A 43-year-old woman, who had previously undergone a right adrenalectomy for primary aldosteronism, was referred for an abnormal heart silhouette on a chest X-ray, which had not been seen three years earlier. Contrast-enhanced computed tomography and coronary angiography (CAG) revealed a giant aneurysm on the anterior aspect of the heart associated with two CPAFs. Because of the risk of rupture of the aneurysm, surgical resection was recommended; however, the patient requested endovascular therapy. On the day of intervention, CAG showed spontaneous occlusion of the feeding vessel to the aneurysm, and the aneurysm showed minimal contrast agent, suggesting spontaneous thrombosis. Because of possible recanalization of the aneurysm, coil embolization was performed, without complications. The patient remained asymptomatic, and the aneurysm was completely embolized at the one-year follow-up.

**Discussion:**

The case shows that minimally invasive endovascular treatment is feasible instead of surgical resection for giant aneurysms associated with CPAFs, depending on their morphological characteristics. This perspective may offer novel insights into treatment strategies for CPAF.

**Learning points**

- Coronary-to-pulmonary artery fistulas complicated by giant aneurysms are rare, and the treatment strategy has not been established.
- Thrombosis of an aneurysm is a result of diverse factors, which include aneurysm morphology, reduced blood flow, vessel wall inflammation, and hypercoagulability.

## Introduction

A coronary artery fistula (CAF) is an abnormal communication between a coronary artery and great vessel or cardiac chamber.^1^ This condition, although rare, has been reported to coexist with aneurysms in 19% of cases.^2^

The case report herein delineates an instance of spontaneous thrombotic occlusion within an aneurysm associated with a coronary-to-pulmonary artery fistula (CPAF), a subtype of CAF. The principal objective of this report was to enhance awareness concerning the anatomical conditions that may predispose a patient to benefit from an endovascular therapeutic modality.

## Summary figure

**Figure:**
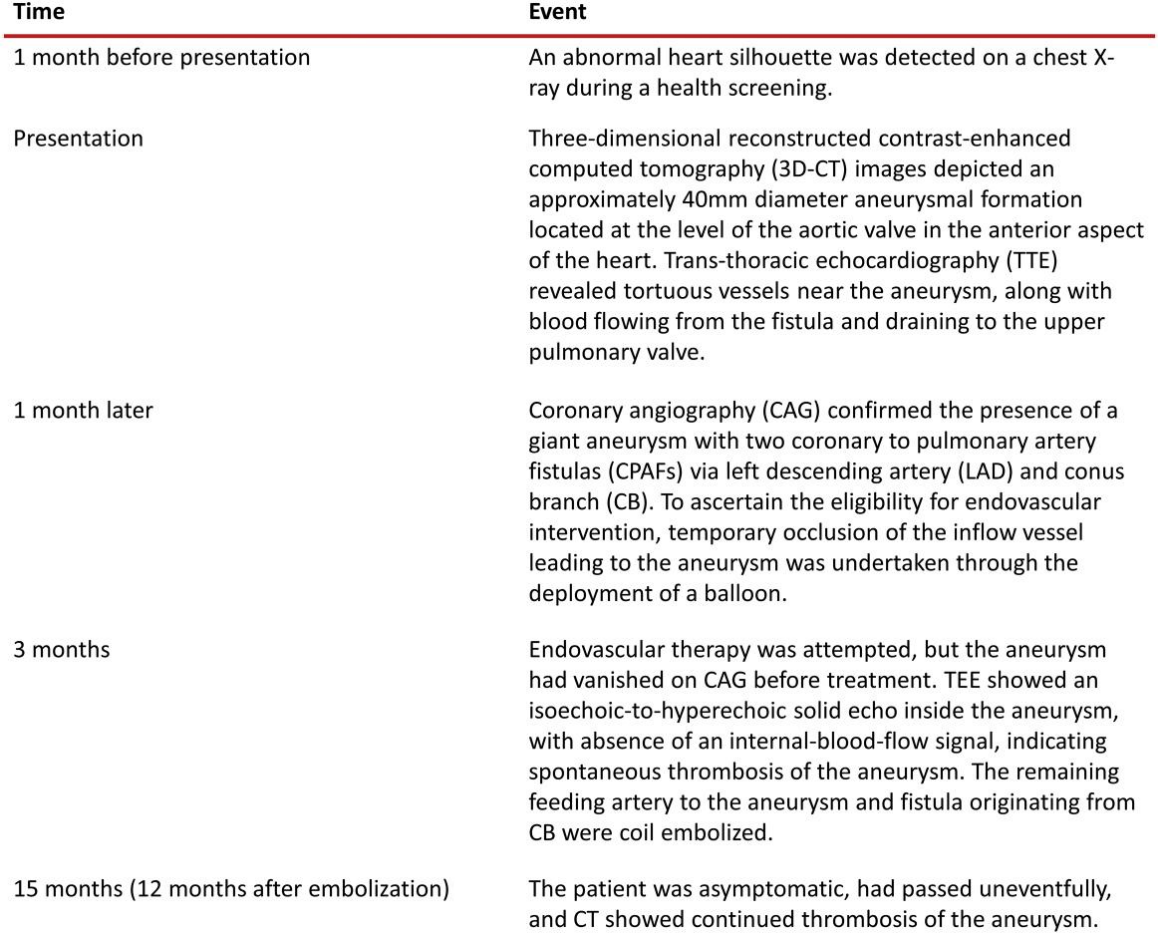


## Case presentation

**Figure 1:**
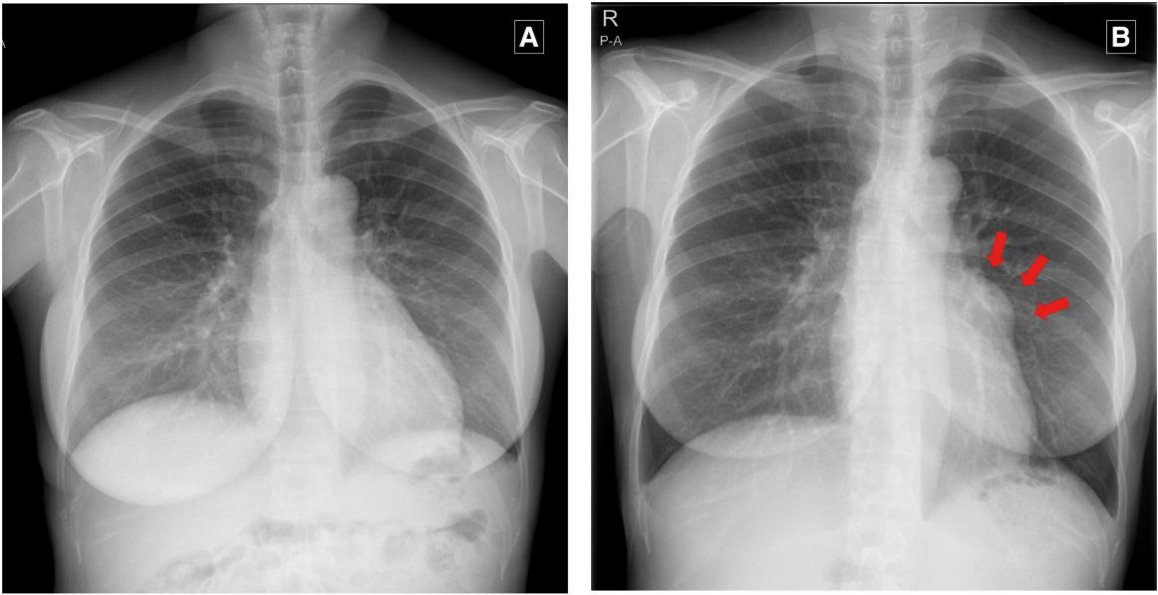
Chest X-ray (*A*) three years previously and (*B*) at presentation. An abnormal silhouette is discernible near the left pulmonary artery (arrows).

A 43-year-old woman first presented to a medical centre after an abnormal heart silhouette was detected on a chest X-ray during a health screening, which had not been present three years earlier (*Figure 1*). Her significant past history included undergoing a right adrenalectomy for primary aldosteronism, with good post-operative results. She was not on any regular medications.

**Figure 2:**
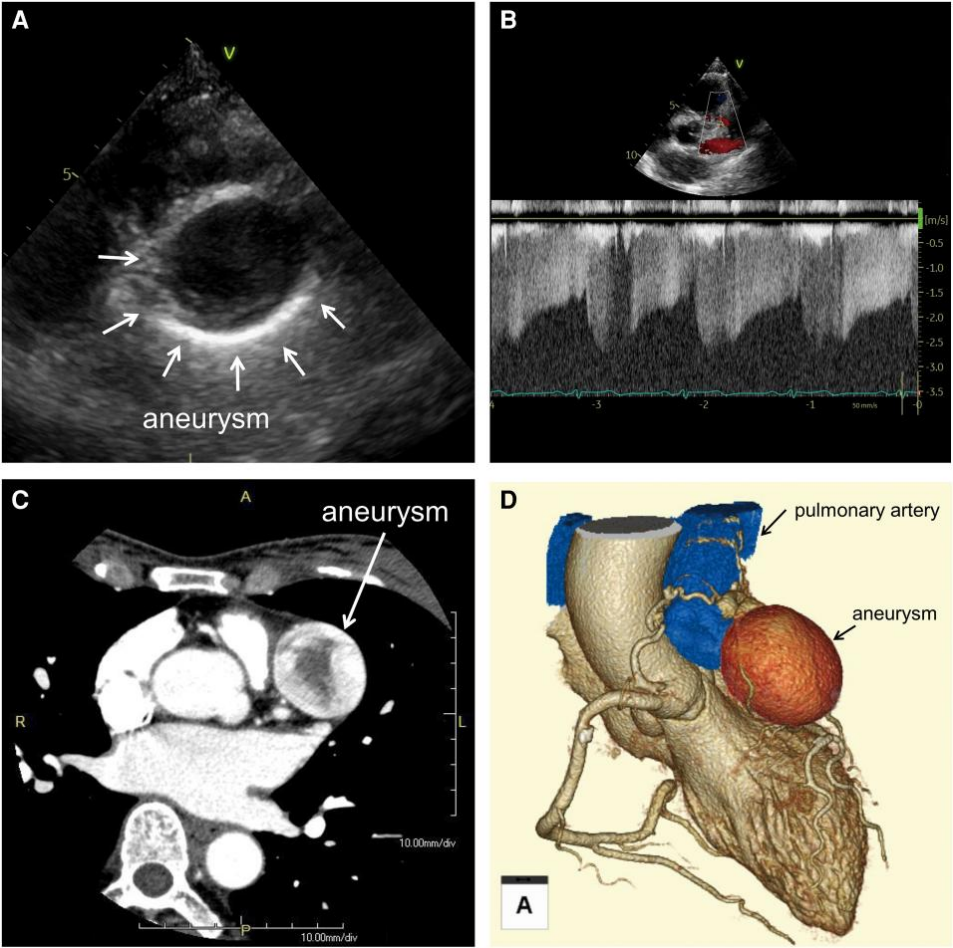
(*A* and *B*) Transthoracic echocardiography images and (*C* and *D*) contrast-enhanced computed tomography images at presentation.

Upon presentation for the chest X-ray findings, the patient was asymptomatic, and the results of physical examination were within normal limits. The blood tests that included d-dimer and immunological assessments were within normal limits. Transthoracic echocardiography (TTE) demonstrated an aneurysmal formation on the epicardial aspect at the base of the anterior wall of the left ventricle (LV) (*Figure 2A*). Tortuous vessels were seen near the aneurysm, along with blood flowing from the fistula and draining to the upper pulmonary valve. These vessels displayed a mosaic signal and a systolic-dominant continuity waveform on Doppler echocardiography (*Figure 2B*). Three-dimensional reconstructed contrast-enhanced computed tomography (3D-CT) images depicted a 43 × 42 × 33 mm aneurysm, located at the level of the aortic valve on the anterior aspect of the heart (*Figure 2C* and *D*). A whole-body CT scan was negative for other aneurysms in other regions of the body. Fluorodeoxyglucose positron emission tomography (FDG-PET)-CT was negative for abnormal uptake in the aneurysm or other organs.

**Figure 3:**
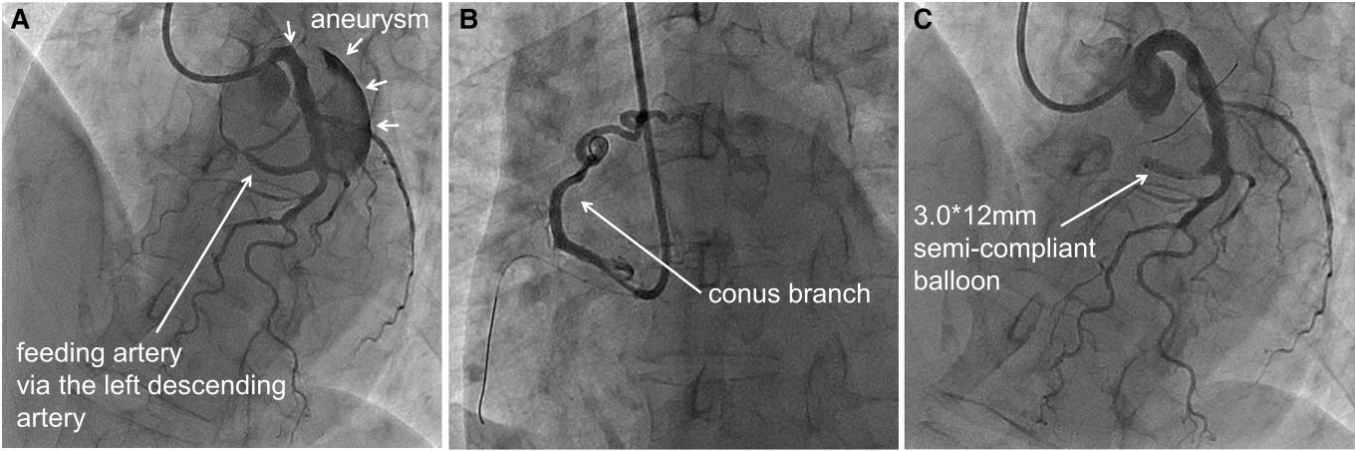
Coronary angiography. (*A*) Left coronary angiography confirmed the presence of a giant aneurysm with coronary-to-pulmonary artery fistula originating from the left descending coronary artery. (*B*) On selective CB angiography, the vessel meandered and culminated leading to the pulmonary artery. (*C*) Experimental occlusion of the feeding vessel using a semi-compliant balloon abolished the inflow of contrast into the aneurysm.

After admission, coronary angiography (CAG) confirmed the presence of two CPAFs via the left anterior descending (LAD) coronary artery (*Figure 3A*, Supplementary material online, *Video 1*) and conus branch (CB) of the right coronary artery (*Figure 3B*, Supplementary material online, *Video S1*), and one giant aneurysm. Selective injection of contrast agent via the LAD revealed inflow of contrast into the aneurysm and outflow into the pulmonary artery (PA). Additionally, experimental balloon occlusion of the feeding vessel abolished inflow of contrast into the aneurysm (*Figure 3C*).

Selective angiography of the CB did not clearly show an inflow into the aneurysm. Right heart catheterization showed a mild increase in oxygen saturation from the right ventricle to the PA (72% and 76%, respectively), with a normal mean PA pressure and PA wedge pressure. The pulmonary-to-systemic flow ratio was 1.00.

An instantaneous wave-free ratio (iFR) measurement was performed in the LAD to confirm the steal due to the CPAF. Marked myocardial ischaemia was not detected (iFR = 0.96: below 0.89 indicate ischaemia). Altogether, the findings were diagnosed as congenital CPAFs with a giant aneurysm.

**Figure 4:**
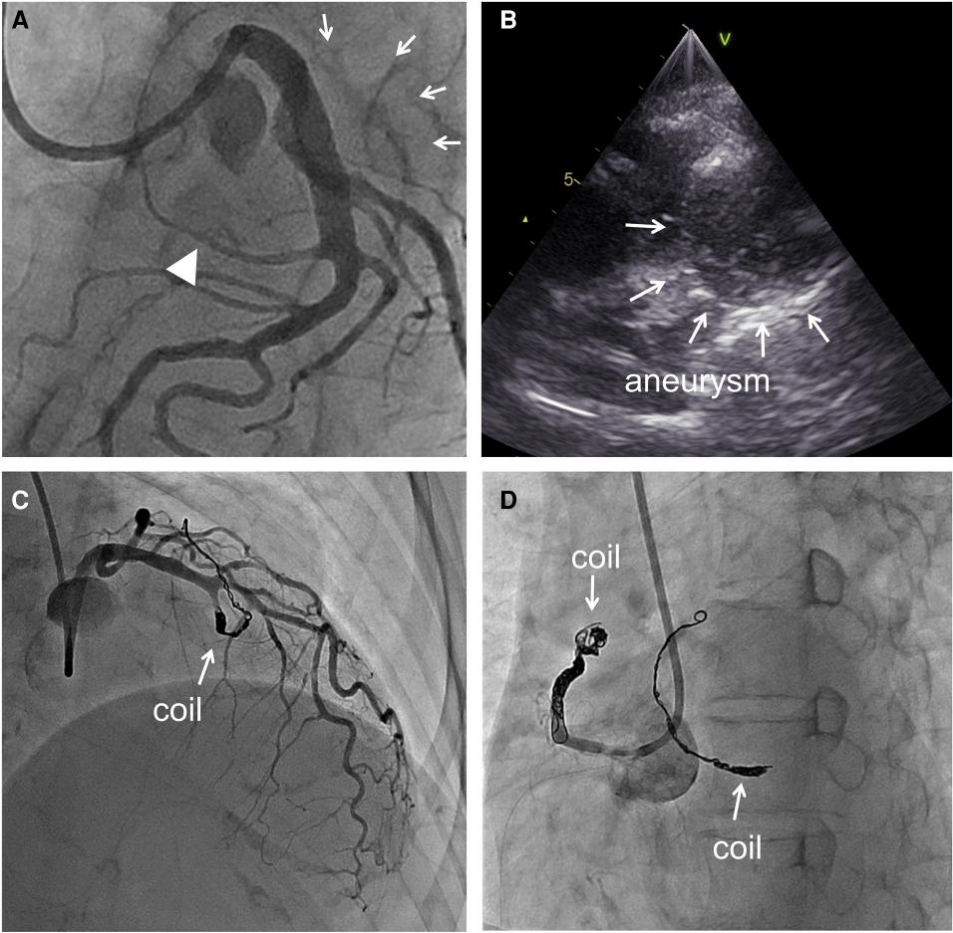
(*A*) Coronary angiography on the day of embolization. The feeding artery via the left descending artery (LAD) was occluded midway (triangle), and the aneurysm showed minimal contrast agent (*B*) Echocardiography on the catheter bed. Isoechoic-to-hyperechoic solid echo inside the aneurysm, and a blood flow signal was absent. (*C*) After embolization of the feeding artery via the LAD. (*D*) After embolization of the coronary-to-pulmonary artery fistula of the conus branch.

Because of the risk of rupture of the aneurysm, which had expanded over a few years, we recommended surgical resection. However, the patient refused this option and opted for endovascular therapy. The patient was prescribed beta-blockers and readmitted for treatment two months later. Her condition was stable, but CAG revealed that the feeding vessel via the LAD was occluded midway, and the aneurysm no longer showed much contrast agent (*Figure 4A*, Supplementary material online, *Video 2*). TTE showed an isoechoic-to-hyperechoic solid echo inside the aneurysm, with absence of an internal-blood flow signal, indicating spontaneous thrombosis of the aneurysm (*Figure 4B*).

**Figure 5:**
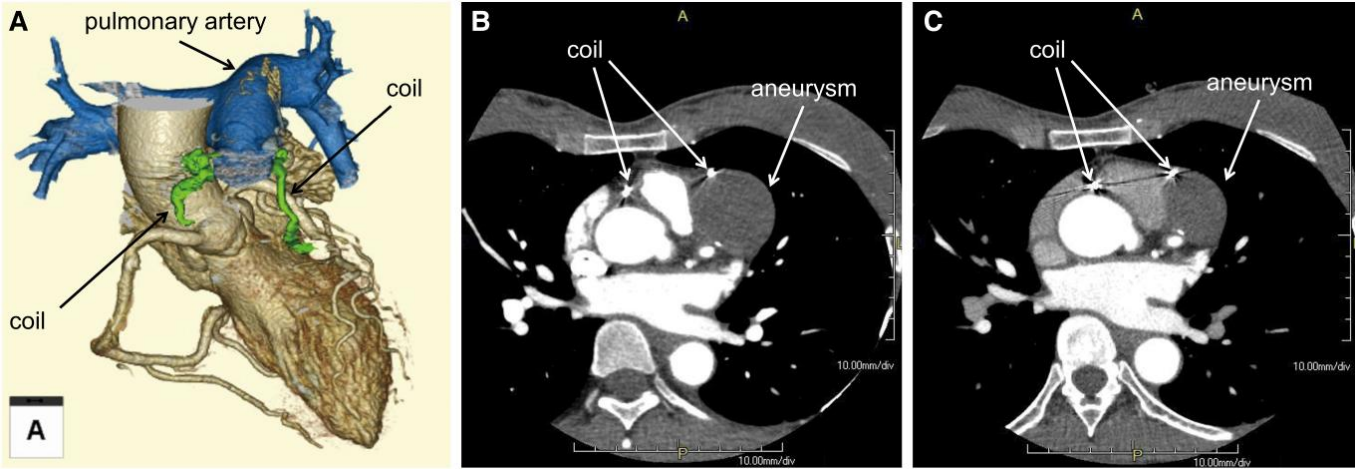
(*A*) Three-dimensional reconstructed contrast-enhanced computed tomography (CT) image after coil embolization. (*B* and *C*) Serial contrast-enhanced CT images, (*B*) 2 days after the procedure, and (*C*) 1 year later.

Since we were concerned about recanalization of the aneurysm, we performed coil embolizations of the feeding vessel in the LAD artery (*Figure 4C*, Supplementary material online, *Video 3*) and the CB (*Figure 4D*, Supplementary material online, *Video S2*), using four 0.018″ straight type coils and nine 0.014″ deatchable coils. Periprocedural complications were not observed. Contrast-enhanced CT conducted on the second day after the procedure showed that the feeding artery was completely embolized by coils and that the aneurysm was almost completely thrombosed (*Figure 5A* and *B*). There was minor residual flow from the draining vessels into the aneurysm. Additionally, there was slight evidence of a fistula connecting the bronchial artery to the CPAF, which had not been discernible on pre-procedural studies. As these findings had no bearing on the patient’s haemodynamic status, we did not perform an additional intervention.

The patient was discharged on the third post-procedural day, free of any complications and without the need for supplementary medication. At a one-year follow-up after the procedure, the patient remained asymptomatic, and a follow-up contrast-enhanced CT was negative for changes. The thrombus inside the aneurysm remained intact (*Figure 5C*).

## Discussion

We treated a patient with an aneurysm larger than 40 mm that was associated with a CPAF. The aneurysm had thrombosed before intervention. The CAF was initially documented in 1865.^3^ It has a reported prevalence in the adult population of 0.1–0.8%.^4^ Aneurysms rarely complicate these conditions, and a rupture is often fatal and includes cardiac tamponade^5^ and sudden death.^6^ Preventative measures include surgical resection^5^ and endovascular embolization.^7,8^ Endovascular therapy, notable for its minimally invasive attributes, is a compelling alternative option. However, owing to the rarity of CAF associated with an aneurysm, the availability of anatomical and morphological references pertinent to endovascular intervention is notably scarce, and current guidelines do not delineate the criteria that define the appropriateness of endovascular treatment for these cases.^9^

Although investigations of the correlation between the morphology of aneurysms and the development of thrombosis have been limited, previous reports have attributed thrombosis of aneurysms of the cerebral arteries to various factors, including aneurysm morphology, decreased blood flow into the aneurysm,^10,11^ inflammatory processes in the vessel wall,^12^ and hypercoagulability.^13^ These findings align with Virchow’s triad that thrombosis is contingent upon the composition of the blood, status of the vascular endothelium, and the specific local haemodynamic conditions.^14^ Samuel *et al*. also reported that thrombosis might occur when the volume of the aneurysm chamber’s volume substantially exceeds the inflow vessels or is considerably narrower.^15^

In this patient, the feeding artery was tapered and markedly narrower than the aneurysm. Furthermore, the balloon occlusion test interrupted the flow of blood into the aneurysm, leading to stasis of the blood flow, which may have promoted thrombosis. The interplay of hydrodynamic factors, affected by the morphological features of the aneurysm; extrinsic perturbations such as inflammatory changes in the feeding vessel as a result of balloon dilation; and the lack of antithrombotic medication may have promoted a coagulation cascade within the aneurysm even after the trial balloon was deflated. The final result was the spontaneous thrombosis of the aneurysm.

## Patient’s perspective

The patient is extremely grateful to be free from the fear of a ruptured aneurysm, and for her physicians’ use of minimally invasive treatment for her condition and for a clinical course without recanalization.

## Conclusion

To our best knowledge, this is the first account of spontaneous thrombosis of a giant aneurysm that was linked to a CPAF. Even giant aneurysms may be suitable for minimally invasive treatment, depending on their morphology.

## Supplementary Material

ytae227_Supplementary_Data

## Data Availability

Data are available upon reasonable request.

## Lead author biography

Tomonori Takahashi, MD, PhD, who is affiliated with Tokushima University Hospital and specializes in the Department of Cardiology, completed his medical school education at Osaka City University Faculty of Medicine and pursued his residency at Matsusaka Municipal Hospital, Kyoto Katsura Hospital in the Department of Cardiology. From 2019 to now, he has conducted research and clinical working at Tokushima University. His primary areas of expertise include interventional cardiology and echocardiography.
